# Impact of diabetic mellitus and cardiac autonomic neuropathy cooccurrence on postinduction hypotension incidence in old patients who underwent general anesthesia

**DOI:** 10.1186/s12877-025-06531-2

**Published:** 2025-11-07

**Authors:** Sirikarn Siripruekpong, Santi Anchalee, Pannawit Benjhawaleemas, Jatuporn Pakpirom, Siriraksa Pooriyapan, Sopida Kampeng

**Affiliations:** Department of Anesthesiology, Faculty of Medicine, Prince of Songkla University, Hat Yai, Songkla, 90110 Thailand

**Keywords:** Diabetes mellitus, Cardiac autonomic neuropathy, Post-induction hypotension, Old patient, Geriatrics, General anesthesia

## Abstract

**Objective:**

Post-induction hypotension (PIH) is defined as mean arterial pressure less than 30% from baseline. It significantly affects patients’ quality of life and can cause morbidity; however, its prognosis remains unclear, and its treatments need improvement. This study aimed to investigate the impact of diabetes mellitus (DM) and cardiac autonomic neuropathy (CAN) co-occurrence on PIH incidence in old patients and evaluate the effects of vasopressor/inotropic drugs on intraoperative complications during post-induction.

**Material and methods:**

This prospective observational study included 92 old patients with DM who planned for elective noncardiac/neuro surgery under general anesthesia. The patients were evaluated with Composite Autonomic Symptom Score 31 (COMPASS31) for CAN preoperatively. During the operation, vital signs were recorded for PIH evaluation.

**Results:**

CAN incidence was 8.70%. PIH incidence in old patients with DM with CAN was 87.5% (vs. DM without CAN 67.9%) (*p* = 0.427). The percentages of patients with DM and CAN and those without CAN were 50% and 38.1% (*p* = 0.707), respectively, in needing a vasopressor drug, and were 87.5% and 75% (*p* = 0.675), respectively, with intraoperative complications.

**Conclusion:**

PIH incidence tended to be higher in old patients with DM and CAN than in those without CAN; however, the difference was not statistically significant. Furthermore, no significant differences were observed between these two groups of patients in using a vasopressor/inotropic drug or having intraoperative complications. However, the blood pressure trend showed more lability during induction and intubation in those with CAN. These findings could help develop new strategies to treat DM, CAN, and PIH.

## Introduction

Post-induction hypotension (PIH) occurs during the first 20 min after anesthesia induction. Its incidence in old patients is 64.8% [1], based on the definition of hypotension in each study. Intraoperative hypotension causes many complications, such as acute stroke [2], acute myocardial infarction [3, 4], and acute kidney injury [4]. The intraoperative period refers to the time between when a patient receives anesthesia and the end of the surgery.

Various definitions of PIH are classified as relative and absolute hypotension thresholds. Examples of relative hypotension thresholds include MAP less than 20% or 30% from baseline blood pressure, and absolute hypotension thresholds include systolic blood pressure (SBP) less than 90 mmHg and MAP less than 65 mmHg.

The risk factors of intraoperative hypotension include patient, anesthetic, and surgical factors. The patient factors are old patients [5, 6], diabetes mellitus (DM) [5], cardiac autonomic neuropathy (CAN) [7], hypertension [5], preoperative blood pressure [6], and American Society of Anesthesiologists (ASA) physical status [6]. The anesthetic factors are fentanyl dosing [6] and propofol induction [6]. The surgical factors include emergency surgery [8]. The complications, such as anaphylaxis, tension pneumothorax, patient positioning, pneumoperitoneum, vagal reflex, massive blood loss, myocardial infarction, septicemia, air embolism, and malignant hyperthermia are the risks of intraoperative hypotension.

Older patients with metabolic conditions like DM, hypertension, and dyslipidemia have a high incidence PIH. CAN is a common DM complication, characterized by impaired cardiovascular autonomic control, with incidence rates ranging from 7.7% to 90% [9]. CAN disrupts hemodynamic stability by affecting heart rate, cardiac output, and blood vessel function. While CAN is linked to poor hemodynamic control, there is no direct data showing that this instability during anesthesia induction increases the risk of complications from anesthetic drugs or airway manipulation.

Several methods are available to assess CAN. The standard options include Ewing’s tests and computerized heart rate variability tests, with high sensitivity (up to 98% and 99%) [10] and specificity (91% and 100%) [11, 12]. Alternative approaches, like the COMPASS31 (Composite Autonomic Symptom Score 31) [13–15] and SAS (Survey of Autonomic Symptoms) [16] questionnaires, offer sensitivities of 77.8% and 93.3% [15], and specificities of 71.7% and 68.3% [16], respectively. However, despite the accuracy of Ewing’s tests, they may pose risks for older patients, such as syncope or heart attack. Studies have shown that patients with diabetes (DM) and CAN, particularly those with orthostatic hypotension, exhibit instability in cerebral blood flow during active standing, indicating impaired cerebral autoregulation [17]. Due to these concerns, this study selected the safer COMPASS31 questionnaire to evaluate CAN.

This study aimed to investigate PIH incidence in old patients with DM and CAN and the effects of vasopressor and inotropic drugs on intraoperative complications during the post-induction period. We hypothesized that older patients with DM and CAN would have a higher PIH incidence than those without CAN. The findings could help inform new treatment strategies for DM, CAN, and PIH.

## Methods

### Study design and setting

This observational study was conducted at Songklanagarind Hospital between June 7, 2020, and March 28, 2022.

### Study participants and study criteria

The study enrolled patients aged 65 and older with ASA physical status II or III who were scheduled for an elective noncardiac surgery and non-neurosurgery under general anesthesia. Exclusion criteria included patients who were admitted to the intensive care unit before surgery, had implantable or external circulatory assist device, had been using a vasopressor or inotropic drug, had poor cardiac function with ejection fraction less than 35%, intubated before surgery, predicted difficult intubation, intubated two or more attempts, planned for the rapid sequence induction technique, planned for combined general anesthesia with regional anesthesia, and participated another study.

### Sample size calculation

The sample size was calculated using a cohort study formula based on research on CAN and abnormal cardiovascular reactions under general anesthesia [7]. With a 5% significance level and 80% power, the required sample size was 16 per group, adjusted to 18 per group to account for a 10% dropout rate, totaling 36 subjects. However, 92 subjects were ultimately included in the study.

### Exposure measurement: DM with/without CAN

The patients were evaluated for CAN using the COMPASS-31 questionnaire [13], which consists of six domains: orthostatic intolerance, vasomotor, secretomotor, gastrointestinal, bladder, and pupillomotor. The total score of all subscales ranges from 0 to 100. Patients were classified into the CAN group if their COMPASS-31 score was 28.6 or higher [15].

### Outcome measurement: postinduction hypotension

The primary outcome was post induction hypotension (PIH) incidence in old patients with DM, without CAN and with CAN. PIH is defined as mean arterial pressure less than 30% from baseline within 20 min after induction. The secondary outcomes were intraoperative vasopressor/inotropic drug use and intraoperative cardiac event. The vital signs: systolic blood pressure, diastolic blood pressure, mean arterial pressure, and heart rate were recorded every minute for the first 20 min following with at least 5 min interval until the end of the operation. The intraoperative cardiac events were noted, such as intraoperative hypotension, intraoperative hypertension, bradycardia, and cardiac arrhythmias.

### Measurement of patient, anesthetic, and surgical factors

The baseline characteristics data were recorded by a researcher, including sex, age, body weight, height, body mass index (BMI), hematocrit, creatinine, ASA Classification, antihyperglycemic drug use, duration of DM, fasting blood sugar, HbA1C, comorbidity disease, current medication, and baselined vital signs at the ward. The site of operation, induction to intubation time, and induction to incision time were recorded.

## Study protocol

The day before surgery, baseline characteristics were recorded, and CAN was evaluated using COMPASS31. Patients did not receive premedication. On the day of surgery, fasting blood sugar was checked, and pre-induction vital signs were recorded. Induction drugs included fentanyl (1–3 mcg/kg IV), propofol (titrated), and cisatracurium (1–1.5 mg/kg IV). General anesthesia was maintained with desflurane, and blood sugar levels were measured hourly until the operation ended. Hypoglycemia and hyperglycemia were managed at the discretion of the attending anesthesiologist.

### Statistical analysis

Categorical variables were reported as frequency and percentages and were compared using Fisher's exact test or Chi-square test as appropriate. All continuous variables were tested for the normal distribution and were presented as median and interquartile range (IQR) or mean and standard deviation (SD). The continuous variables were analyzed using the Student’s t-test or Wilcoxon Rank-sum test as appropriate. A *P*-value of less than 0.05 was considered statistical significance. All statistical analyses were performed using the R program.

### Ethical considerations

Ethical approval for this study was obtained from the Office of Human Research Ethics Committee, Faculty of Medicine, Prince of Songkla University, on 27 May 2020 (REC 62–192-8–1). All participants received an explanation of the study protocol, and informed consent was obtained from them.

## Results

**Fig. 1:**
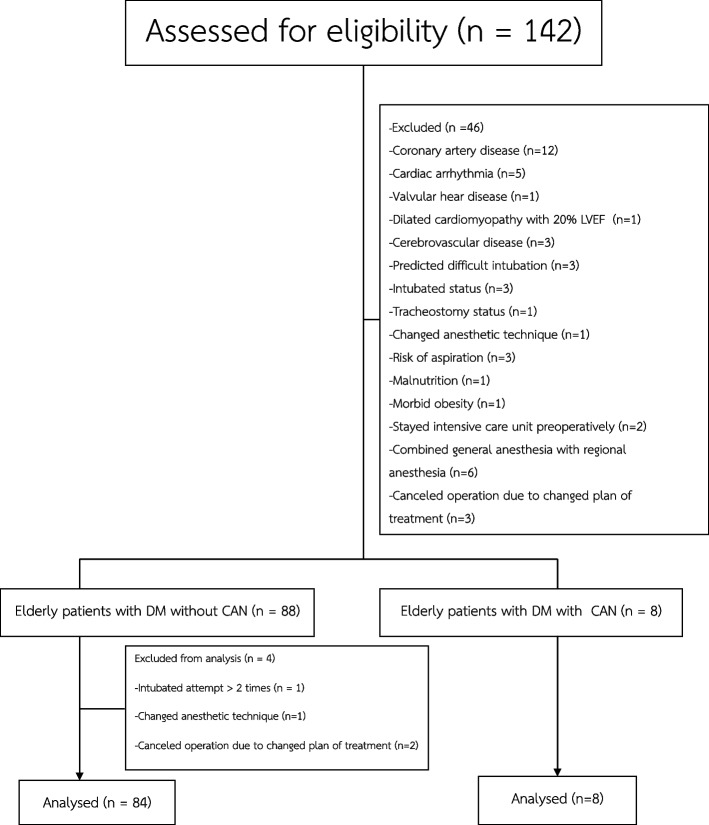
Study consort flowchart

From June 7, 2020, to March 28, 2022, 142 participants were screened for eligibility, and 96 participants were enrolled in this study. Four participants were excluded from the analysis; thus, 92 participants were enrolled in the study (Fig. 1).

CAN incidence in old patients with DM was 8.70%. Patients with CAN had higher body weight (69.8 ± 12.4 vs. 61.9 ± 10.5) (*p* = 0.047), higher BMI (27.8 ± 5.9 vs. 24.9 ± 3.7) (*p* = 0.046), lower hematocrit (30.5 ± 4.4 vs. 36.2 ± 4.6) (*p* = 0.001), higher creatinine (1.4 (1.2,1.4) vs. 0.9 (0.7,1.2)) (*p* = 0.003), higher usage of insulin (50% vs. 11.9%) (*p* = 0.017), longer duration of DM (18.5(15,25.8) vs. 10 (4,15)) (*p* = 0.006), and more usage of angiotensin receptor blocker (ARB) (62.5% vs. 13.1%) (*p* = 0.004) than those without CAN (Table 1).

**Table 1:** Baseline demographic data and outcomes

	Older adults with DM without CAN (*n* = 84)	Older adults with DM with definite CAN (*n* = 8)	*P*-value
Sex; Male, No. (%)	35 (41.7)	2 (25)	0.468*
Age (year), Median (IQR)	70 (67,75.2)	68 (65.8,72.2)	0.37†
Body weight (kg), Mean (SD)	61.9 (10.5)	69.8 (12.4)	0.047‡
Height (cm), Mean (SD)	157.6 (9.1)	158.9 (5.5)	0.672‡
BMI (kg/m^2^), Mean (SD)	24.9 (3.7)	27.8 (5.9)	0.046‡
Hematocrit, Mean (SD)	36.2 (4.6)	30.5 (4.4)	0.001‡
Creatinine, Median (IQR)	0.9 (0.7,1.2)	1.4 (1.2,1.4)	0.003†
ASA Classification, No. (%)			0.481*
Class II	44 (52.4)	3 (37.5)	
Class III	40 (47.6)	5 (62.5)	
Diabetes mellitus			
Insulin use, No. (%)	10 (11.9)	4 (50)	0.017*
Duration of DM (year), Median (IQR)	10 (4,15)	18.5 (15,25.8)	0.006†
Fasting blood sugar (mg%), Median (IQR)	132 (114,151.5)	133.5 (127.2,153.2)	0.598†
HbA1C, Median (IQR)	6.9 (6.2,8)	6.6 (5.8,7.4)	0.369†
Comorbidity disease, No. (%)	82 (97.6)	8 (100)	1*
Coronary artery disease	1 (1.2)	1 (12.5)	
Cerebrovascular disease	7 (8.3)	1 (12.5)	
Peripheral vascular disease	1 (1.2)	0 (0)	
Hypertension	69 (82.1)	8 (100)	
Dyslipidemia	62 (73.8)	7 (87.5)	
End stage renal disease	3 (3.6)	0 (0)	
Hyperthyroidism	0 (0)	1 (12.5)	
Cancer	24 (28.6)	2 (25)	
COPD	3 (3.6)	0 (0)	
Current medications, No. (%)	68 (81)	8 (100)	0.342*
Beta blocker	16 (19)	1 (12.5)	1*
Calcium channel blocker	36 (42.9)	3 (37.5)	1*
ACEI	24 (28.6)	3 (37.5)	0.689*
Angiotensin receptor blocker	11 (13.1)	5 (62.5)	0.004*
Diuretics	10 (11.9)	1 (12.5)	1*
Vasodilator	13 (15.5)	2 (25)	0.612*
Thyroid therapy	0 (0)	1 (12.5)	0.087*
COMPASS31 score, Median (IQR)	13 (8.8,17.8)	30 (29,32.2)	< 0.001*
Average baseline vital signs			
Systolic BP (mmHg), Mean (SD)	135.5 (16.4)	140.1 (14.1)	0.402‡
Diastolic BP (mmHg), Mean (SD)	69.8 (5.9)	73.5 (7.6)	0.205‡
MAP (mmHg), Mean (SD)	92.8 (7)	95.9 (8.2)	0.335‡
Heart rate (beats/min), Mean (SD)	71 (6.8)	74.8 (11.7)	0.401‡
Anesthetic drug			
Propofol (mg), Median (IQR)	100 (80,120)	100 (87.5,105)	0.581†
Fentanyl (mcg), Median (IQR)	100 (75,100)	100 (93.8,100)	0.902†
Cisatracurium (mg), Median (IQR)	10 (8,10)	10 (10,10)	0.266†
Induction to intubation time (mins)	8 (5,10)	10 (8.5,12)	0.073‡
Induction to incision time (mins)	25 (20,34.2)	30 (15.5,36.2)	0.95†
Operation time (mins)	165 (125,232.5)	205 (113.8,258.8)	0.613†

*BMI* body mass index, *COPD* chronic obstructive pulmonary disease, *ACEI* angiotensin-converting enzyme inhibitor, *COMPASS31* composite autonomic symptom scale 31

†Wilcoxon Rank-sum test

‡Student’s t-test

Data analysis: * Fisher's exact test

The overall PIH incidence in patients in this study was 69.57%. No difference in PIH incidence was observed in patients with and without CAN in both the absolute hypotension threshold, SBP less than 90 mmHg (62.5% vs. 53.6%) (*p* = 0.723), MAP less than 65 mmHg (62.5% vs. 63.1%) (*p* = 1) and the relative hypotension threshold, MAP less than 30% baseline (87.5% vs. 67.9%) (*p* = 0.427). The incidences of inotropic/vasopressor drug use during the post-induction period (50% vs. 38.1%) (*p* = 0.707) and intraoperative cardiac event (87.5% vs. 75%) (*p* = 0.675) were not different in both groups (Table 2).

**Table 2:** Incidences of post-induction hypotension, inotropic and vasopressor drug use, and intraoperative complications

Post-induction hypotension	Older adults with DM without CAN (*n* = 84)	Older adults with DM with CAN (*n* = 8)	*P*-value
Primary outcome			
Postinduction hypotension	57 (67.9)	7 (87.5)	0.427*
Secondary outcome			
Inotropic/vasopressor drug during post-induction period	32 (38.1)	4 (50)	0.707*
Inotropic/vasopressor drug during intraoperative period	41 (48.8)	6 (75)	0.268*
Postinduction hypotension with absolute hypotension threshold			
SBP < 90 mmHg, No. (%)	45 (53.6)	5 (62.5)	0.723*
MAP < 65 mmHg, No. (%)	53 (63.1)	5 (62.5)	1*
Intraoperative cardiac event, No. (%)	63 (75)	7 (87.5)	0.675*

Intraoperative hypertension was defined as SBP > 180 mmHg

*DM *Diabetes Mellitus*, CAN *Cardiac Autonomic Neuropathy*, SBP *Systolic blood pressure*, MAP *Mean arterial pressure

Data analysis: *Fisher's exact test

**Fig. 2:**
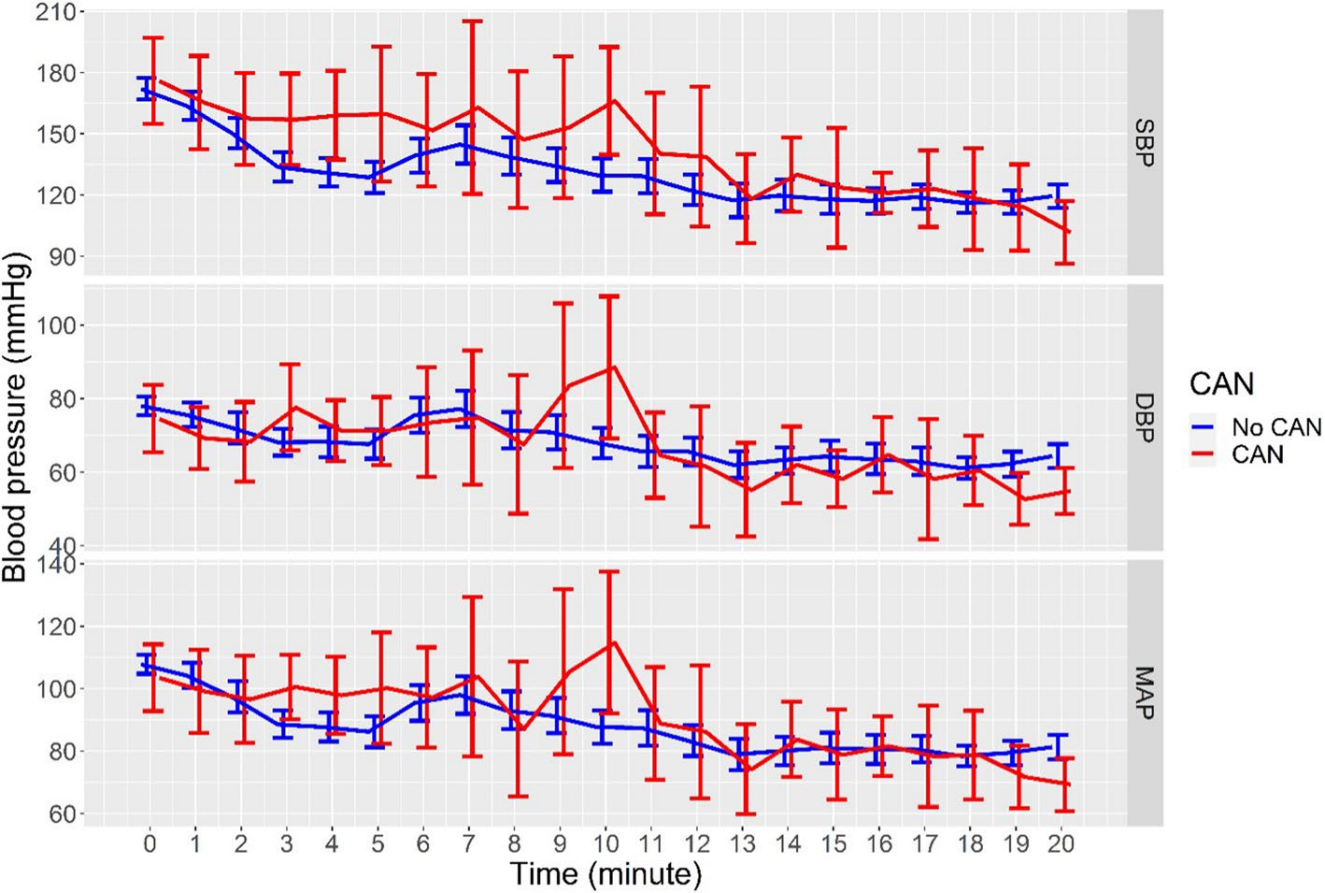
Systolic blood pressure, diastolic blood pressure, and mean arterial pressure in the post-induction period. (mean ± standard error)

**Fig. 3:**
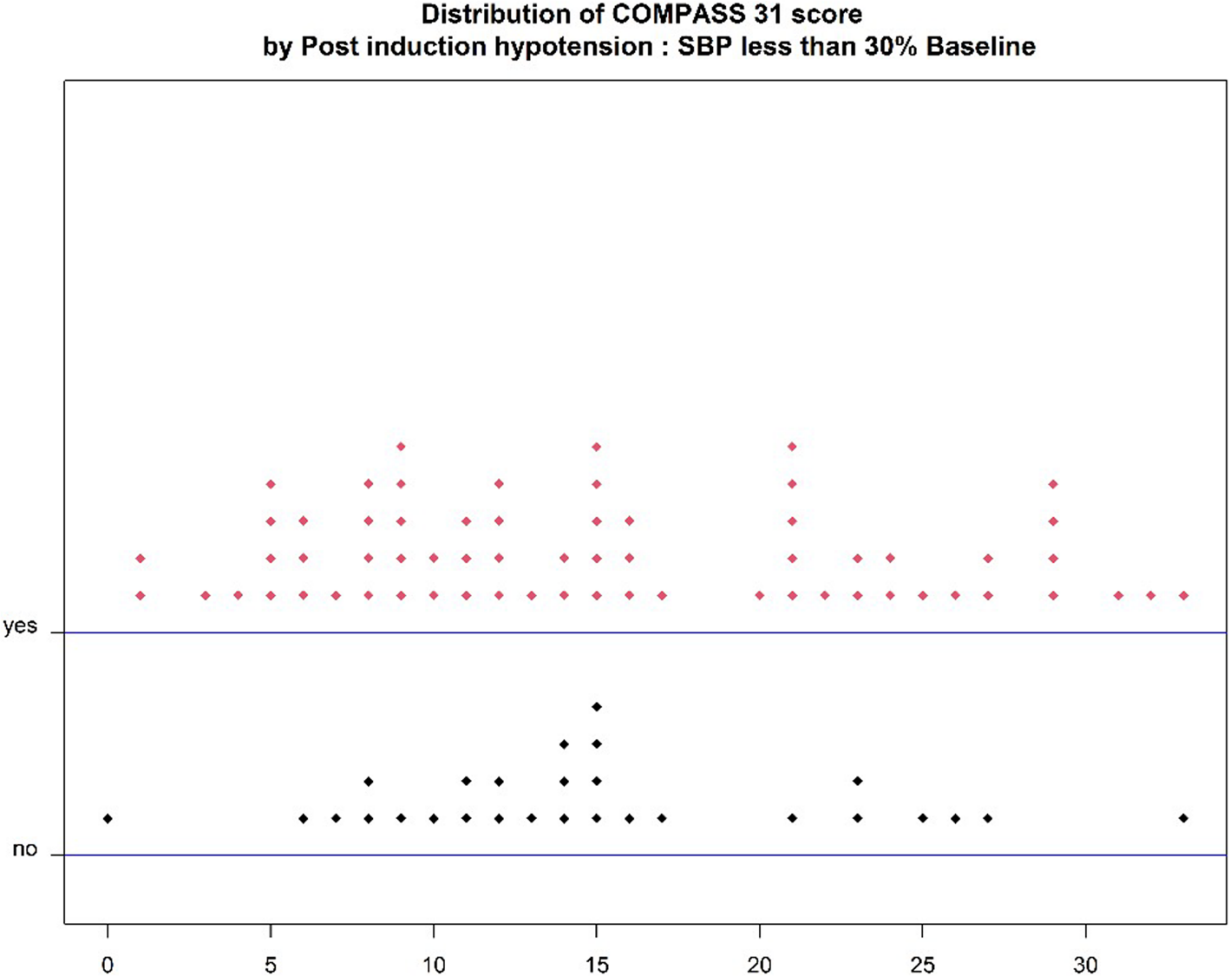
Distribution of COMPASS31 scores by post-induction hypotension

The trends of the systolic blood pressure during the first 20 min of the operation, the diastolic blood pressure, and the mean arterial pressure during the induction period in patients with and without CAN were various. After induction with anesthetic agents, SBP, DBP, and MAP trended to decrease from baseline in the first three minutes. At the time of intubation, blood pressure trended to rise, and the group with CAN showed more increase in blood pressure than the group without CAN. Therefore, the group with CAN trended to have decreased blood pressure again and receded more than the group without after intubation and while waiting for the surgical incision (Fig. 2). The COMPASS31 scores in the group with PIH compared with the group with post-induction normotension were not different (*p* = 0.862) (Fig. 3).

## Discussion

The incidence of CAN in our study of older patients with diabetes (DM) was 8.70%. The prevalence of CAN in various studies ranges from 7.7% to 90% [9]. CAN is classified by severity into early, definite, and severe categories using standard tests such as Ewing’s tests [10]. A COMPASS31 score cutoff of 28.67 points can be used to distinguish patients with definite CAN from those without it [15]. However, some patients with early CAN may not be distinguishable from those without CAN using the COMPASS31 score.

The overall incidence of PIH in our study was 69.57%. A previous study found that intraoperative hypotension occurred in 28.6% of patients with DM without CAN and 72.2% in those with both DM and CAN (*p* < 0.05). Our research focused on patients over 65, as aging is a known risk factor for intraoperative hypotension. Consequently, CAN may not significantly contribute to the high PIH incidence in older patients, who are already at risk due to age. In clinical practice, the PIH incidence in older patients with DM and CAN was notably high at 87.5%. Therefore, anesthesiologists should closely monitor this patient group during general anesthesia, ensuring optimized preoperative evaluations and preparations to manage potential hemodynamic instability during the induction period.

After anesthesia induction, the blood pressure tended to decrease in the first three minutes and increase at the time of intubation. After intubation, patients with CAN tended to exhibit more decreased blood pressure than those without CAN. In consequence of trend of blood pressure in CAN group seemed to be labile more than no CAN group, the anesthesiologist should be concern to additional techniques that could be controlled the blood pressure during the airway manipulation and also beware of receding the blood pressure after intubation until surgical stimulation occurred.

To reduce postinduction hypotension, anesthesiologists may consider using etomidate instead of propofol, as previous study indicate that propofol is an independent predictor of hypotension after induction [6], while etomidate is associated with a lower incidence of intraoperative hypotension [18]. Another approach involves a lidocaine-based induction regimen in older patients, which has been shown to reduce post-induction hypotension compared to fentanyl-based regimens [19]. Additionally, total intravenous anesthesia with remimazolam, a novel short-acting benzodiazepine, has been associated with less intraoperative hypotension than propofol in older patients, with similar outcomes in recovery time, delirium, and postoperative nausea and vomiting [20].

### Limitation

In our study we used COMPASS31 to evaluated the CAN condition, for questionnaire technique has some limitation in older patient because they might be not clearly remember of recall or remote information. The other concern of research is the COMPASS31 is not a standard test for CAN. The standard tests for this condition are the Ewing tests and computerized heart rate variability test. Ewing test has much higher both the sensitivity and specificity than COMPASS31 [10]. COMPASS31, with cutoff point 28.6, can classify definite CAN and no CAN [10] but cannot divide the CAN into groups as early, definite, and severe CAN; this might be due to the fact that CAMPASS31 cannot be used to distinguish patients with early CAN from those without CAN.

Finally, the small number of subjects allocated to the CAN group in our study may have affected the power of our findings. A future study with a larger sample size is encouraged to improve the reliability of the results.

## Conclusions

The incidence of PIH was higher in older patients with DM and CAN compared to those without CAN, though the difference was not statistically significant. Similarly, the incidence of intraoperative complications from vasopressor/inotropic use showed no significant difference between the two groups. CAN incidence in older patients with DM was lower than in previous studies, possibly due to the use of COMPASS31 for assessment. Anesthesiologists managing older patients with DM and CAN should carefully select appropriate drugs and interventions to reduce the high incidence of PIH in this group.

## Data Availability

We already uploaded raw data of the research for submission's process.

## Abbreviations

ACEI: Angiotensin converting enzyme inhibitor
ANS: Autonomic Nervous System
BMI: Body Mass Index
CAN: Cardiac autonomic neuropathy
COMPASS31: Composite Autonomic Symptom Score 31
COPD: Chronic obstructive pulmonary disease
DBP: Diastolic blood pressure
DM: Diabetes Mellitus
GA: General anesthesia
IQR: Interquartile range
IV: Intravenous
LVEF: Left ventricular ejection fraction
MAP: Mean arterial pressure
PIH: Postinduction hypotension
PNS: Parasympathetic Nervous System
SAS: Survey of Autonomic Symptoms
SBP: Systolic blood pressure
SD: Standard deviation
SE: Standard error
SNS: Sympathetic Nervous System
SAS score: Survey of Autonomic Symptoms score
